# Supplementary figures and images for: Rate-of-Kill (RoK) assays to triage large compound sets for Chagas disease drug discovery: Application to GSK Chagas Box
Source: PLoS Negl Trop Dis. 2021 Jul 16;15(7):e0009602. doi: 10.1371/journal.pntd.0009602 (PMC8318231; doi:10.1371/journal.pntd.0009602)

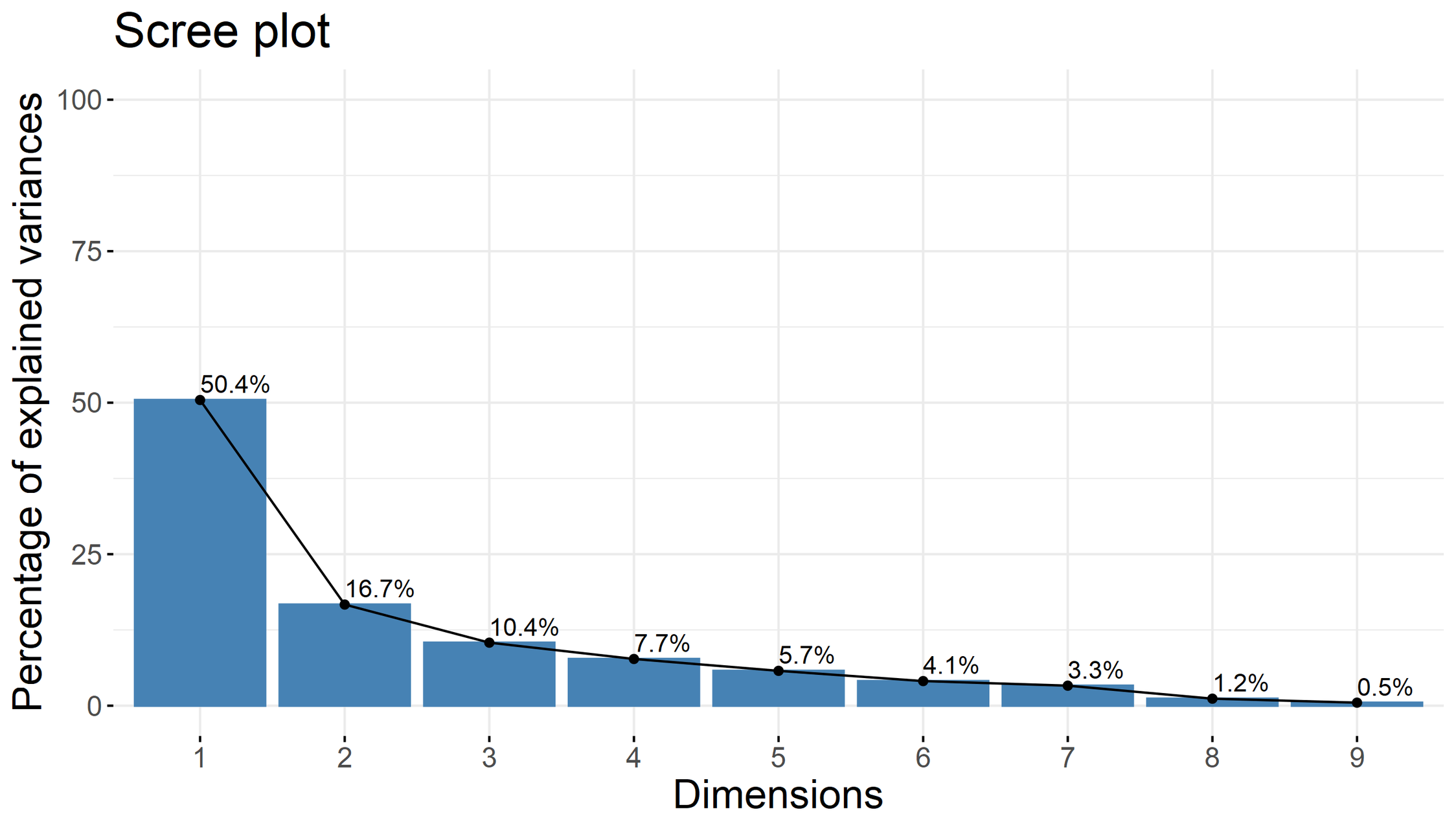

Supplement: S1 Fig — Percentage of variance explanation for each dimension labels each bar. Dimension 1 and 2 covering 67% variance have been selected for further analysis. (TIF) [file pntd.0009602.s003.tif]

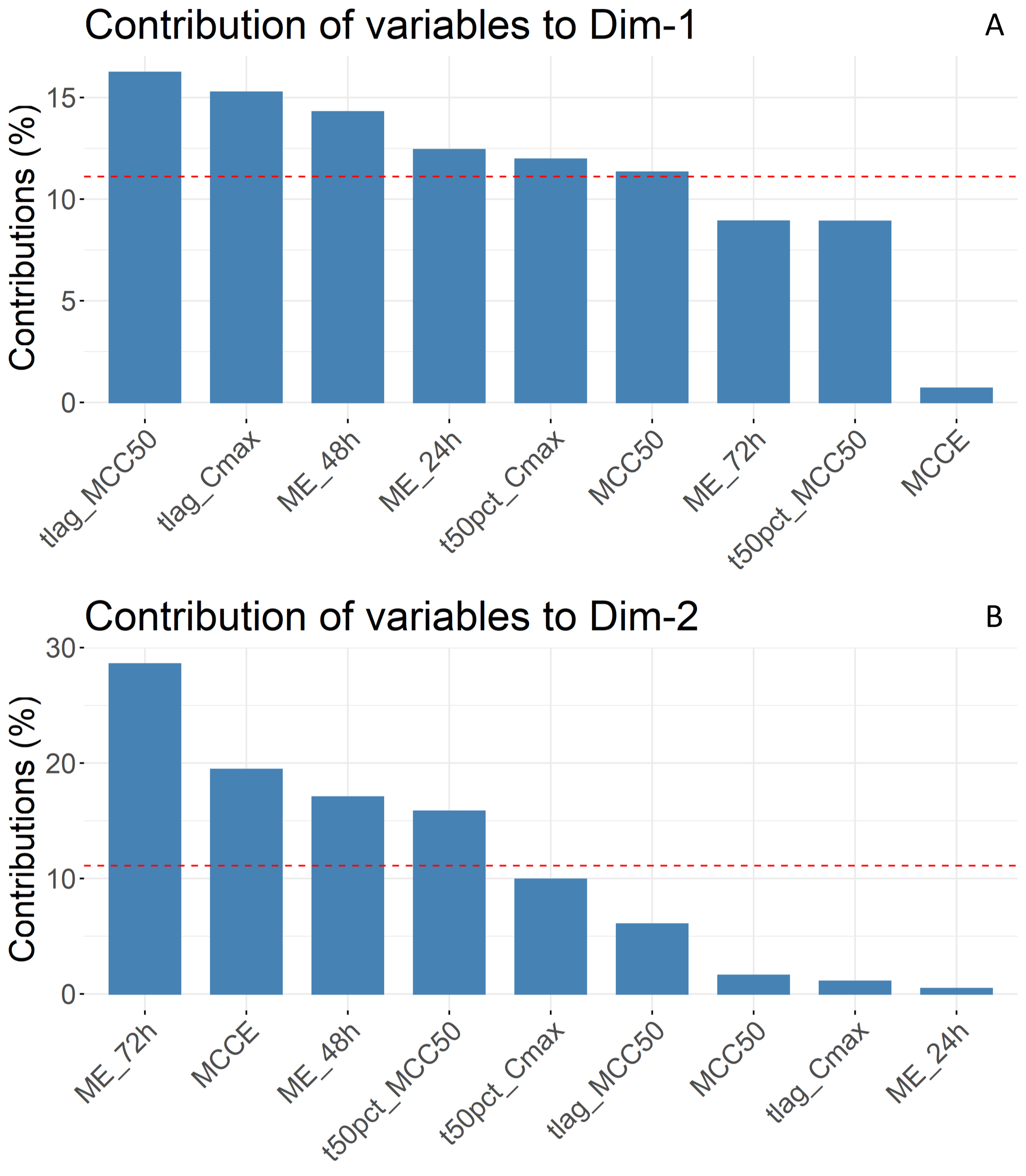

Supplement: S2 Fig — (TIF) [file pntd.0009602.s004.tif]

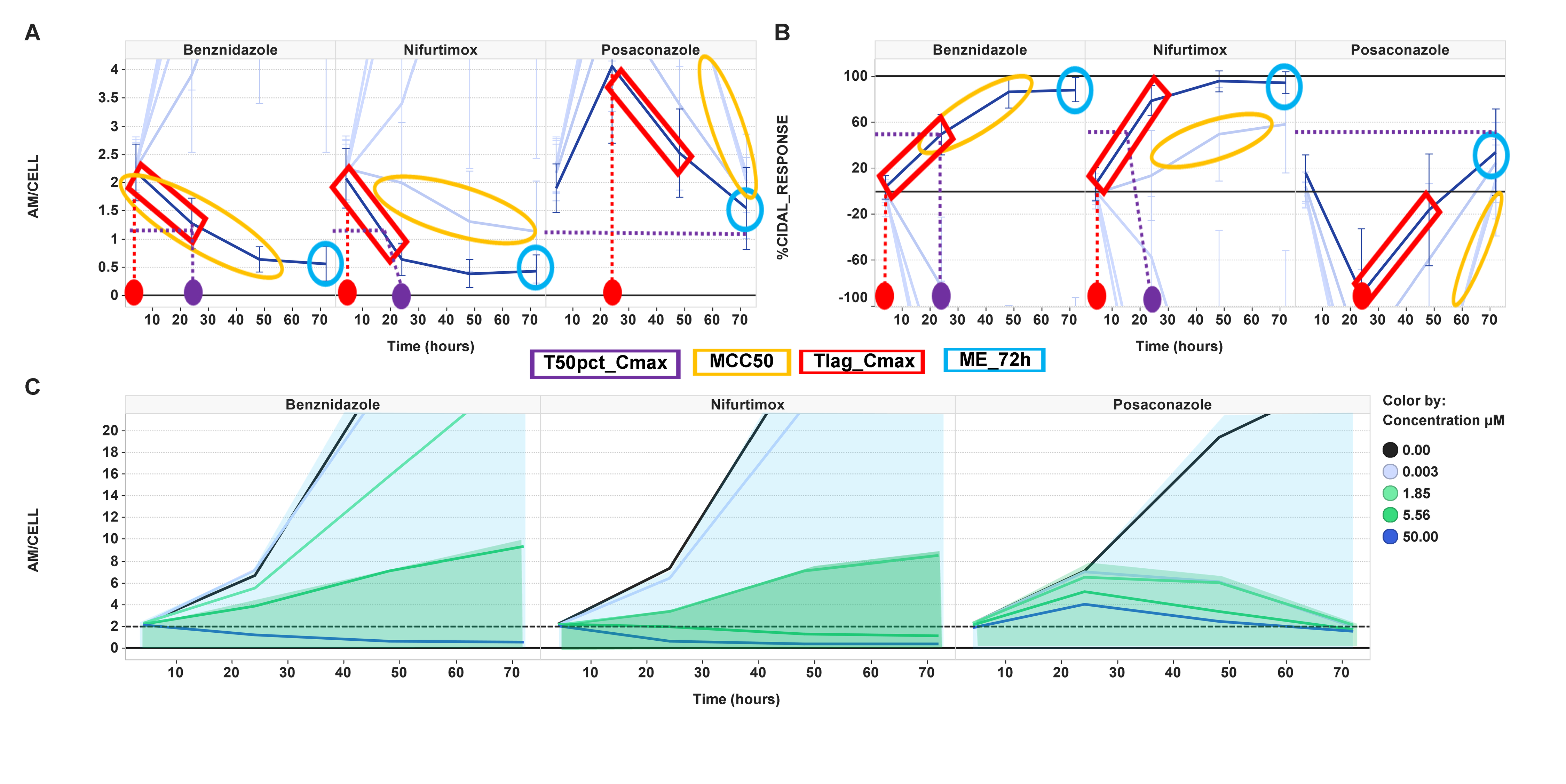

Supplement: S3 Fig — (A-B) Data of Fig 2 were used to illustrate the RoK curve descriptors. In blue ME_72h, in red Tlag_Cmax, in yellow MCC50 and in purple T50pct_Cmax. Definition of all RoK curve descriptors are included in Methods section of the manuscript. (C) For MCCE schema non-normalised data (i.e. average of amastigotes per cell) were used Area in blue corresponds to the AUC of control sample in the absence of compound. The time-course line in green corresponds to the lowest compound concentration that reduces by 50% the AUC of control sample, i.e. MCCE. AUC for MCEE is coloured in light green. (TIF) [file pntd.0009602.s005.tif]

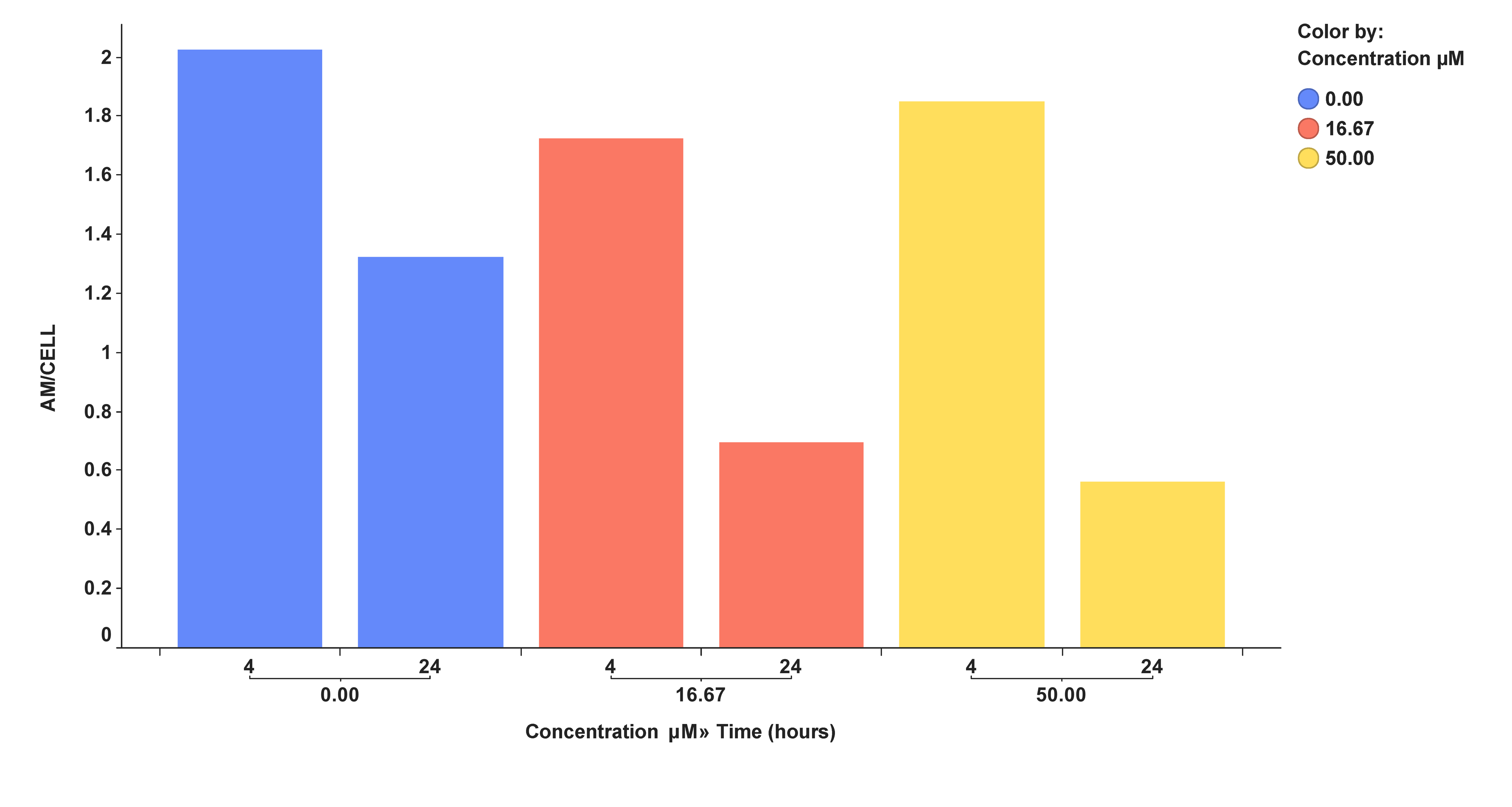

Supplement: S4 Fig — Average of T. cruzi amastigotes per cell was plotted in Y axis and bars were coloured by concentration of compound 1 (50 μM in yellow, 16.67 μM in red and no compound in blue). 50 μM BNZ was present in all the cases. Two time-points were represented, i.e. 4 and 24 hours. (TIF) [file pntd.0009602.s006.tif]
