# Supplementary material for: Rate-of-Kill (RoK) assays to triage large compound sets for Chagas disease drug discovery: Application to GSK Chagas Box
Source: PLoS Negl Trop Dis. 2021 Jul 16;15(7):e0009602. doi: 10.1371/journal.pntd.0009602 (PMC8318231; doi:10.1371/journal.pntd.0009602)
Supplement: Attachment — Submitted filename: Responses to Editors and Reviewers_v2.pdf [file pntd.0009602.s009.pdf]

Helton da Costa Santiago, M.D., Ph.D
Associate Editor
PLOS Neglected Tropical Diseases

Helen Price
Deputy Editor
PLOS Neglected Tropical Diseases

Madrid, 11th June 2021

On behalf of all the authors of the PNTD-D-21-00399 manuscript entitled *"Rate-of-Kill (RoK) assays to triage large compound sets for Chagas Disease drug discovery: Application to GSK Chagas Box",* we appreciate your consideration of its publication upon thorough revision following feedback from you and the reviewers. We thank for all the comments, suggestions, and questions. They are fair and thoughtful, and priceless to improve the quality of the paper. We have done our best to meet all their points. You can find our 22 responses interspersed along your letter below, each of the responses being adjacent (and in green) to each of the points made by the reviewers.

Looking forward to our manuscript being eventually approved.

Yours sincerely

Julio Martin, PhD
Freelance
Sciengement Lab Consulting

Decision on your PLOS Neglected Tropical Diseases submission (PNTD-D-21-00399) - [EMID:258ecfff87ef332e]

"Rate-of-Kill (RoK) assays to triage large compound sets for Chagas Disease drug discovery: Application to GSK Chagas Box"

Dear Dr Martin,

Thank you very much for submitting your manuscript "Rate-of-Kill (RoK) assays to triage large compound sets for Chagas Disease drug discovery: Application to GSK Chagas Box" for consideration at PLOS Neglected Tropical Diseases. As with all papers reviewed by the journal, your manuscript was reviewed by members of the editorial board and by several independent reviewers. In light of the reviews (below this email), we would like to invite the resubmission of a significantly-revised version that takes into account the reviewers' comments.

The manuscript was reviewed by experts in the field which agreed on its importance. However, the reviewers feel that there is space for much improvement regarding to concepts, as pointed by reviewer #3, and coherence, as pointed by reviewer #1, must be addressed by the authors. Improvement of methodological description and clarification of apparent results discrepancies need to be addressed by the authors. The submission of the program coding (if not protected by patent request) would be of great interest by the public.

- **Response #1:** We are providing the program coding in R language. It is written as ASCII file, so that it can be open and edited as text file (e.g., with Notepad), and can be run in R or RStudio.

We cannot make any decision about publication until we have seen the revised manuscript and your response to the reviewers' comments. Your revised manuscript is also likely to be sent to reviewers for further evaluation.

When you are ready to resubmit, please upload the following:

[1] A letter containing a detailed list of your responses to the review comments and a description of the changes you have made in the manuscript. Please note while forming your response, if your article is accepted, you may have the opportunity to make the peer review history publicly available. The record will include editor decision letters (with reviews) and your responses to reviewer comments. If eligible, we will contact you to opt in or out.

[2] Two versions of the revised manuscript: one with either highlights or tracked changes denoting where the text has been changed; the other a clean version (uploaded as the manuscript file).

Important additional instructions are given below your reviewer comments.

Please prepare and submit your revised manuscript within 60 days. If you anticipate any delay, please let us know the expected resubmission date by replying to this email. Please note that revised manuscripts received after the 60-day due date may require evaluation and peer review similar to newly submitted manuscripts.

Thank you again for your submission. We hope that our editorial process has been constructive so far, and we welcome your feedback at any time. Please don't hesitate to contact us if you have any questions or comments.

Sincerely,

Helton da Costa Santiago, M.D., Ph.D
Associate Editor
PLOS Neglected Tropical Diseases

Helen Price
Deputy Editor
PLOS Neglected Tropical Diseases

****

The manuscript was reviewed by experts in the field which agreed on its importance. However, the reviewers feel that there is space for much improvement regarding to concepts, as pointed by reviewer #3, and coherence, as pointed by reviewer #1, must be addressed by the authors. Improvement of methodological description and clarification of apparent results discrepancies need to be addressed by the authors. The submission of the program coding (if not protected by patent request) would be of great interest by the public.

Reviewer's Responses to Questions

Key Review Criteria Required for Acceptance?</br></br> As you describe the new analyses required for acceptance, please consider the following:</br></br>

Methods</br></br>
-Are the objectives of the study clearly articulated with a clear testable hypothesis stated?</br> -Is the study design appropriate to address the stated objectives?</br> -Is the population clearly described and appropriate for the hypothesis being tested?</br> -Is the sample size sufficient to ensure adequate power to address the hypothesis being tested?</br> -Were correct statistical analysis used to support conclusions?</br> -Are there concerns about ethical or regulatory requirements being met?</br></br>

Reviewer #1: See below

Reviewer #2: Most results are shown as averages and in some cases (Fig. 6C) error bars are shown but their meaning is not indicated. It would be important to have a section on Statistical analyses.

- **Response #2**: We have added details on statistical analysis at the legends to figures, wherever appropriate (e.g., Fig. 2 and 6C)

Reviewer #3: This manuscript describes the results of study aimed at proposing rate-of-kill (RoK) assays as a method to triage potential drug candidates for the etiological treatment of American Trypanosomiasis (Chagas disease, CD), a systemic parasitosis caused by the kinetoplastid parasite Trypanosoma cruzi. The aim is certainly of interest, as CD is the largest parasite burden in the American continent, now spreading to non-endemic areas due to international migrations and leading to severe cardiac and/or GI tract physiopathological alterations in ca. 30% patients. However, it is one of the most neglected diseases in the world as specific chemotherapy of this

condition is very unsatisfactory, particularly in its prevalent chronic stage: the only drugs currently available in clinical settings, the nitroheterocyclic compounds benznidazole and nifurtimox, have limited and variable efficacy and frequent adverse effects, leading to treatment discontinuation in 10-30% of patients. As a result, it is currently estimated that <1% of patients receive treatment for this condition.
Unfortunately, the motivation of the study is seriously questionable as its central premise is that fast activity (high RoK) is the key property to be prioritized in the development of anti-T. cruzi drugs (see page 6 and the Discussion), although it is well known that such compounds (including benznidazole and nifurtimox) are characterized by unspecific cytotoxicity that naturally leads to low selectivity and toxic side effects, in vitro and in vivo. Fast acting compounds typically act by what is called a Cmax effect, based on reaching a threshold concentration at the target organisms that triggers a rapid breakdown of essential structures and functions and cell death, while slow acting compounds act by specifically interfering with essential metabolic pathways in the target and require a sustained exposure, thus their activity is associated to the area under the curve (AUC; concentration vs time) of the drug, and **are typically of low toxicity;** for a recent experimental demonstration and discussion of this fundamental difference between the two types of drugs, see **Meyer et al. (2019). J Antimicrob Chemother 2019; 74: 2303–2310**.

- **Response #3:** The authors agree on this classification of drugs in general terms. Nevertheless, we think that it cannot be assumed that all fast-acting drugs based on Cmax must be toxic compounds for the host cells. Safety is a complex multifactorial outcome derived from combined features, such as **(i) specificity of the mode of action** (i.e. single target vs. promiscuity, off-target effects and poly-pharmacology), **(ii) selectivity** (i.e. higher molecular recognition of the parasite target over the counterpart host target), **(iii) PK/PD at the site of action** (i.e. proper pharmacokinetics may result into pharmacological effects on the parasite, but not on the host, just as Meyer et al. describes in their paper), **(iv) selective activation and uptake mechanisms at the parasite** (i.e. even if the molecular target is present in both parasite and host cells, the drug may require specific biological systems for its activation, such as nitroreductases for nitro-aromatic compounds, or its intracellular transport) **and (v) cellular plasticity** (i.e. even if the compound could also recognise the molecular target at the host, these cells may activate compensation mechanisms to elude this effect, which might not be present in a biologically simpler organism as the parasite is). Actually, and in alignment with these reviewer´s comments and the paper, what we propose is that fast acting compounds will be **more toxic ONLY for the parasite**, and that it is possible to find out such compounds which can exquisitely differentiate parasite from host cells, thus worth pursuing effort. Hence, we seek compounds which resemble toxic profile of nitroaromatic compounds (such as NIF and BNZ), **BUT ONLY against trypanosomes, thus being INACTIVE against host cells**, based on any or several of the aforementioned mechanisms.

Results</br></br>
-Does the analysis presented match the analysis plan?</br> -Are the results clearly and completely presented?</br> -Are the figures (Tables, Images) of sufficient quality for clarity?</br></br>

Reviewer #1: See below

Reviewer #2: Specific comments:

Line 76: a reference is needed concerning nitroreductases.

- **Response#4**: The following reference has been added.

Stephen Patterson and Susan Wyllie (2014) Nitro drugs for the treatment of trypanosomatid diseases: past, present, and future prospects. Trends in Parasitology 30 (6); Open AccessPublished:April 26, 2014 DOI:https://doi.org/10.1016/j.pt.2014.04.003

Line 89: a reference (Urbina, J. Eukaryot. Microbiol. 2015) suggested that those disappointing results were due to the use of posaconazole at a dose that was just 10-20% of that measured in mice at the curative dose of 20 mg/kg/d.

- **Response #5**: Thanks for making the point. We unintentionally overlooked this hypothesis. We agree with the reviewer and have added reference and comment in the Introduction (line 90): "…(4, 7-9). Some authors have pointed to an inadequate dosing of Posaconazole (i.e. 10-20% of curative does in mice of 20 mg/Kg/d) as the reason for failure". Likewise, we are now including this argument in the Discussion section (lines 492-496).

Line 477: "arguable within the research community". A reference should be added or the sentence should be deleted.

- **Response #6**: Reference 17 is inserted in the text (line 507; please note that line numbering has now changed in the new revised version).

Line 479: A better term than metabolically "poor" should be used or the term deleted. Although they do not divide, trypomastigotes are metabolically very active.

- **Response #7**: We agree. We have deleted and amended accordingly: "Trypomastigotes cultured in vitro are ~~a metabolically poor and~~ non-dividing parasite form possessing a differentiated metabolic stage,…" (current line 509).

Reviewer #3: Yes.

Conclusions</br></br>
-Are the conclusions supported by the data presented?</br> -Are the limitations of analysis clearly described?</br> -Do the authors discuss how these data can be helpful to advance our understanding of the topic under study?</br> -Is public health relevance addressed?</br></br>

Reviewer #1: See below

Reviewer #2: Yes

Reviewer #3: In addition to the considerations presented above (Methods), the authors selectively cite the available scientific literature when claiming that ergosterol biosynthesis inhibitors, which act by selectively interfering with the synthesis of 24-alkyl sterols in fungi and protozoa such as T. cruzi and all species of Leishmania and thus are slow acting (AUC-depending) drugs, by claiming that lack of clinical efficacy of such compounds in recent clinical trials (page 5, refs. 4, 7-9) is associated to their mechanism of action, when the most plausible explanation for these disappointing results is the suboptimal doses and/or treatment duration used in these studies for the anti-T. cruzi indication (see Molina et al. 2015, Curr Opin Infect Dis 28:5, 397-407; Urbina, J.A. 2017. J Am Coll Cardiol. 11;70(2):299-300; Urbina, J.A. 2018. Lancet Infect Dis. 18(4):363-365).

Thus, although the automated methodology described by the authors can nicely cluster separately fast acting from slow acting compounds, which correspond to Cmax (unspecific) and AUC (specific) agents (Fig. 3C and Results, pages 22-24), it is not a foregone conclusion that the former should be prioritized for further experimental and clinical development. On the contrary, such methodology could be very useful for an early characterization of potential candidates for anti-T. cruzi drugs with unknown mechanism of action as fast acting (Cmax) or slow acting (AUC) compounds, and thus raise red flags for potential in vivo toxicity and avoid long and expensive preclinical work with no clinical future.

- **Response #8**: The authors agree with the reviewer on that we had overlooked referencing the drug sub-dosing of Posaconazole as one of the causes of clinical failure. In the revised version we have added this explanation and included citations (e.g. lines 90 and 492). Nevertheless, it is our opinion that explanation of the failure of POS in clinical trials remain to be conclusively unveiled. Likely, there is not a single cause, but a combination of those. Our hypothesis is that compounds exhibiting a fast cidal in vitro behaviour will have a better probability of success in efficacy models. This explanation is also considered by Molina et al (2015) in reference #12: *"Another interesting issue is that, even though ergosterol biosynthesis inhibitors are more potent than nitroheterocyclic compounds, the time needed to reach the same efficacy against T. cruzi is much longer (slow trypanocidal compounds)"*. Therefore, and by exploiting the aforementioned biological mechanisms in reply to reviewer #3, we believe that it is possible to find compounds resembling the in vitro trypanocidal pattern of nitroaromatic drugs, but with superior safety profile. Other explanations pointed out in the literature:
  (1) Benznidazole efficacy seems to be enhanced by **interplaying with immune system**, whereas POS is less dependent on host´s immune system.
  (2) **The animal model itself.** The murine chronic model has been the most used to evaluate the antichagasic candidate molecules, though life expectancy and immune system differ from human beings. The period for initiating therapy after the infection in the chronic model ranges from 45 to 130 days. Considering the life expectancy of mice, this period of infection is still far from the stage of infection found in the clinical practice, wherein the median age of the patients treated is near 40 years old. Thus, the murine in vivo model might overestimate the effect of the drugs. Patients treated with POS, regardless of the dose, had a high treatment failure ratio (80–92.3% of treatment failure). These results are consistent with those from the nonhuman primate model wherein all the animals treated with POS had treatment failure during the follow-up.
  (3) **The mechanism of action (MoA) of azoles makes POS hardly effective versus trypomastigote parasite forms.** The activity of ergosterol biosynthesis inhibitors is time-dependent and results from the depletion of ergosterol pools, since *T. cruzi* is completely dependent on the endogenous synthetised sterols which are crucial for cell division and growth. Those parasitic forms with reduced metabolism activity (quiescent forms) or nondividing stages as trypomastigotes could have a reduced susceptibility to such drugs.

Editorial and Data Presentation Modifications?</br>
 Use this section for editorial suggestions as well as relatively minor modifications of existing data that would enhance clarity. If the only modifications needed are minor and/or editorial, you may wish to recommend "Minor Revision" or "Accept".

Reviewer #1: (No Response)

Reviewer #2: Minor revision

Reviewer #3: OK.

Summary and General Comments</br></br> Use this section to provide overall comments, discuss strengths/weaknesses of the study, novelty, significance, general execution and scholarship. You may also include additional comments for the author, including concerns about dual publication, research ethics, or publication ethics. If requesting major revision, please articulate the new experiments that are needed.

Reviewer #1: In this manuscript, Cantizani et al develop quantitative metrics to identify cidal compounds against T. cruzi, in a format that is suitable for high-throughput analyses. This is a very important issue, with interesting new ways to visualize the data. Considerations of applicability to HTS are also welcome. However, I have the following major concerns:

1. MCC50 is defined by the authors as the lowest concentration that "reduces 50% of the parasite load measured at zero time." However, POS is listed as having an MCC50 of 6.2 uM in Figure 2C, even though in Figure 2A there are no concentrations at which POS reduces parasite load by half.
    - **Response #9:** We thank the reviewer for the fair and thorough observations. We embrace rigour; thus we apologise for the mistake. The confusion stemmed from the fact that the Figure 2A was plotted for just one representative experiment, whereas the values in Figure 2C correspond to the average of several independent experiments. Figure 2A has now been modified including the average and standard deviation of all experiments for each compound concentration. The total number of experiments of each compound included in the calculations and plots is described at the legend of the table in Fig. 2C, and in brackets. Legend of Fig. 2 has been changed to explain the modifications.

2. Likewise, I am puzzled as to how POS attains such a small MCCE given that it never reduces parasite load below ~1.5 and cidal response does not exceed 40%
    - **Response #10:** We understand you can struggle definition of some curve descriptors. Definition of MCCE is not so evident, and we recognize that its meaning may not be clearly captured in the name and acronym. As defined in METHODS section, MCCE is the lowest compound concentration that decreases the AUC by at least 50% with respect to the control in the absence of control (i.e. infected cells), irrespectively of whether the compound is static or cidal. Since POS is a very potent compound, yet static, its calculated MCCE is exceptionally low. Based on your comments, we have realized that a better name for the MCCE acronym should be adopted, and we are renaming MCCE as "Minimal Compound Concentration with Effect". We have accordingly amended the text in the line 210 of METHODS section. Likewise, we have included the supplemental S5 Fig. with a schematic of the RoK curve descriptors. In particular, panel C for MCCE, where you can see the kinetic curve for 0.003 µM POS, precisely its average MCCE, reducing by 50% the AUC of control sample.

3. MCCE for BNZ is calculated as 3.9 uM. I don't understand how such a concentration could be considered to take 46.2 hours to clear 50% of parasites (t50%_MCC50), given that only the highest benznidazole concentration reduces amastigotes/cell compared to the starting load of ~2 ama/cell and other concentrations flatline or are associated with parasite growth in panel 2A.

- **Response #11**: Ditto, in point #2 and response #10 above. We are afraid that the confusion comes up again from the misunderstanding on MCCE meaning, due to our former poor definition. MCCE contemplates effect on the decrease of parasite burden over untreated control, regardless the compound is static or cidal. Instead, MCC50 is the right descriptor to assess compound cidality. For BNZ, MCC50 is 31.2 µM, which is the concentration to collate for t50%_MCC50. We have included the aforementioned supplemental S5 Fig. with a schematic of the RoK curve descriptors, which we hope it is helping to clarify the meaning of the descriptors.

4. t50%_Cmax is defined as the time needed for the highest concentration (50 uM) to reduce parasite load by half. Such a reduction (to ~1 ama/cell) seems to be attained before 20 h for nifurtimox, even though 24h is listed in Figure 2C. Likewise, I don't understand how 65.9 is returned for this value for posa, given that the highest posa concentration doesn't appear to attain a 50% reduction in parasite load even by experiment endpoint.

- **Response #12**: As described in METHODS section, t50%_Cmax for each experimental RoK curve is calculated without interpolation between 4 and 24 hours. Thus, the program renders only discrete values of 4, 24, 48 or 72 hours. We have amended METHODS section at the aim of explaining it more clearly. Please bear in mind that table in Fig. 2C corresponds to average values of each descriptor, thus it may contain intermediate figures. Figures 2A and 2B have been reformatted to include average and standard deviations from independent experiments. Please notice that POS does attain a 50% reduction for some experiments, despite the average does not appear to reach the 50% threshold. Nevertheless, experiments whose descriptors are higher than the maximum experimental concentration and time are disregarded from the average calculation (see Fig 2C legend).

5. Benznidazole at the highest concentration always shows reduction in amastigote numbers at each timepoint. Why then is the tlag so high? I would expect it to be 0 at Cmax.

- **Response #13**: As abovementioned, tlag is not calculated by extrapolation, so that the lowest value is 4 h, which is the first experimental point included in the calculations. Please notice that table in Fig. 2C corresponds to average values of each descriptor, thus it may contain intermediate figures. At least BZN showed a tlag of 24 hours, thus increasing the average value above 4 h.

6. Nifurtimox is shown to clear parasites much faster than benznidazole based on Figure 2 curves; however, the difference in tlag between benznidazole and nifurtimox is quite small. tlag metrics should be improved or modified to reflect this difference.

- **Response #14**: Just for clarification, tlag is not quantifying the steepness of the slope, but the time delayed until the slope of the normalized time-course becomes positive. Thus, tlag is not a direct estimator of the speed of action, but of the time lag spent until the compound starts to reduce the parasite burden. The speed of the killing effect is estimated by the combination of tlag and t50%. As abovementioned, tlag is not interpolated. Both BNZ and NIF exhibit tlag values of either 4 or 24 hours in all the experiments tested, so that the average is an intermediate figure. Lowest tlag is 4 hours, since that is the shortest experimental incubation time.

7. These issues might be addressed by providing a schematic showing exactly how each value is derived from the am/cell and % cidal response curves.

- **Response #15**: Thanks for the advice. A schematic of the RoK curve descriptors has been added as Supplemental Information S5 Fig.

8. Authors laudably talk about the usefulness of this method for HTS. The authors should provide their R script as supplemental data or as a github page. Otherwise, this method will not have any utility beyond the authors' laboratory.

- **Response #16:** We are providing the R script as supplemental information S7. It is written as ASCII file, so that it can be open and edited as text file (e.g. with Notepad), and run in R or RStudio.

9. Lines 391-392, authors state "On one hand, all compounds belonging to the CYP51/azole class are scattered throughout clusters 1, 3, 4 and 5." Close examination of cluster 2 also reveals some pale blue lines. Please clarify.

- **Response #17**: Thanks for the fair observation. It is true that NOT ALL, but MOST OF the compounds (82%) belonging to the CYP51/azole class are scattered throughout clusters 1, 3, 4 and 5. A minority of putative CYP51/azole class (18%) also fall into cluster 2. We have amended the text accordingly (current line 404).

10. Figure 6B: "ellipse in red comprises compounds with activity versus trypomastigotes but inactive against intracellular amastigotes." Why is this an ellipse and not a rectangular box? How were the margins selected?

- **Response #18:** The ellipse represents just a qualitative and arbitrary boundary to illustrate that this class of compounds are detected (former lines 584-585, current lines 616-617). We have amended the legend to reflect this point (line 457). Hard margins and filters can be set at convenience of each researcher depending on the purpose of the selection to push compounds forward.

11. Lines 470-471: "In the case of 10 µM of BNZ, the combination (solid line in blue) allows to reduce the concentration of BNZ by 5-fold to exert the same effect than 50 µM (dotted line in blue).". I am puzzled by this statement. In figure 6C, the combination is showing much superior reduction in ama/cell compared to benznidazole alone, rather than showing the same effect. Please clarify.

- **Response #19:** Apologies for the confusing statement. We were comparing with 50 µM BNZ alone, which is not shown in the Fig 6C. Following reviewer´s request in "minor issue #3" below, we are including these data as supplemental in the bar chart of S6 Fig.

Minor issues:

1. Line 132 typo: "sodiumpyruvate" should be "sodium pyruvate".
- **Response #19:** Corrected.

2. Figure 3C and 4A legends indicates "Color by TARGET". However, the detailed legend is instead a mixture of compound name/class (e.g. NIF, nitroaromatic) and target (e.g. proteasome). Please make consistent.
- **Response #20:** We agree with the reviewer. Fig. 3C has been edited and avoided the colour code for BNZ, NIF and POS, since they are differentiated by shape (as in Fig. 3B). As for Fig. 4A, since shape code is not feasible, we have edited the legend to explain the colour code for BNZ, NIF and POS.

3. Line 470 data not shown: please provide in supplemental
- **Response #21**: Done. A new bar graph has been added as supporting information S6 Fig.

Reviewer #2: This is an interesting work proposing that speed of action and cidality, rather than potency, are better predictors of efficacy of compounds in animal studies, as suggested by previous results with azoles and nitroaromatic compounds. The method described could also be useful to triage large compound sets. This is a thoughtfully considered and well executed study. Overall, this manuscript advances the field significantly and I have only minor suggestions.

Reviewer #3: Based on the previous considerations, it is the opinion of this reviewer that the work is a very valuable effort in advancing the quest for safer and more efficacious chemotherapeutic anti-T. cruzi agents but the premise that originally motivated the study and the discussion of the experimental results presented in the manuscript must be profoundly revised, both on theoretical grounds and for eventual clinical applications of the methodology.

- **Response #22**: We much appreciate the recommendations by reviewer #3. We recognise that we had overlooked to mention the sub-dosing of Posaconazole as a plausible cause of its failure in clinical trials. It was not our intention to rule out this explanation at all. We have edited the introduction section and included that and other references. Likewise, we might have overrated the hypothesis of fast-acting and cidal compounds having a better prospect for translation of in vitro activity into therapeutic efficacy. We agree on not being a foregone conclusion, and recognize there may be different, but compatible, schools of thought. It was not our aim to be exclusive of other approximations. Thus, we have edited the manuscript in order to fairly frame our approach within a general and inclusive context (for instance lines 90 and 492-503). What we propose in this manuscript is an experimental methodology for triaging this kind of compounds as a way of increasing the probability of success towards further efficacy models, providing they have the right PK/PD and ADME-TOX properties. Needless to say, each researcher will eventually have to make his/her own bet and manage the risk.

PLOS authors have the option to publish the peer review history of their article (what does this mean?). If published, this will include your full peer review and any attached files.

If you choose “no”, your identity will remain anonymous but your review may still be made public.

Do you want your identity to be public for this peer review? For information about this choice, including consent withdrawal, please see our Privacy Policy.

Reviewer #1: No

Reviewer #2: No

Reviewer #3: Yes: Julio A. Urbina

Figure Files:
While revising your submission, please upload your figure files to the Preflight Analysis and Conversion Engine (PACE) digital diagnostic tool, https://pacev2.apexcovantage.com. PACE helps ensure that figures meet PLOS requirements. To use PACE, you must first register as a user. Then,

login and navigate to the UPLOAD tab, where you will find detailed instructions on how to use the tool. If you encounter any issues or have any questions when using PACE, please email us at figures@plos.org.

Data Requirements:
Please note that, as a condition of publication, PLOS' data policy requires that you make available all data used to draw the conclusions outlined in your manuscript. Data must be deposited in an appropriate repository, included within the body of the manuscript, or uploaded as supporting information. This includes all numerical values that were used to generate graphs, histograms etc.. For an example see here:
http://www.plosbiology.org/article/info%3Adoi%2F10.1371%2Fjournal.pbio.1001908#s5.

Reproducibility:
To enhance the reproducibility of your results, we recommend that you deposit your laboratory protocols in protocols.io, where a protocol can be assigned its own identifier (DOI) such that it can be cited independently in the future. Additionally, PLOS ONE offers an option to publish peer-reviewed clinical study protocols. Read more information on sharing protocols at
https://plos.org/protocols?utm_medium=editorial-email&utm_source=authorletters&utm_campaign=protocols

---

In compliance with data protection regulations, you may request that we remove your personal registration details at any time. (Use the following URL: https://www.editorialmanager.com/pntd/login.asp?a=r). Please contact the publication office if you have any questions.
